# Single-center results from the first 100 robotic ureteral reimplantation in children: analysis of learning curve effects

**DOI:** 10.3389/fsurg.2025.1681854

**Published:** 2025-10-21

**Authors:** G. Mattioli, G. Brenco, F. Fanti, G. Rotondi, E. Verrina, G. Piaggio, M. B. Damasio, M. Carlucci, V. Fiorenza

**Affiliations:** 1 Pediatric Surgery Unit, IRCCS Istituto Giannina Gaslini, Genoa, Italy; 2 Department of Neuroscience, Rehabilitation, Ophthalmology, Genetics and Maternal Child Science, University of Genoa, DINOGMI, Genoa, Italy; 3 Department of Nephrology Dialysis and Transplant, IRCCS Istituto Giannina Gaslini, Genoa, Italy; 4 Department of Radiology, IRCCS Istituto Giannina Gaslini, Genoa, Italy

**Keywords:** robotic surgery, ureteral reimplantation, learning curve, pediatric robotic surgery, uretero-vesical junction

## Abstract

**Introduction:**

Robot-assisted laparoscopic ureteral reimplantation (RALUR) is increasingly utilized in pediatric urology, yet outcomes vary widely and the learning curve remains under-investigated. This study aims to evaluate perioperative outcomes and learning curve progression during the first 100 pediatric RALUR procedures performed by a single surgeon.

**Methods:**

A prospective, single-center study was conducted on 100 RALUR procedures in 96 pediatric patients between May 2020 and May 2025. The cohort was divided into two groups (first 50 cases vs. second 50) to assess the impact of surgical experience on outcomes. Surgical techniques included both dismembered (D-RALUR) and non-dismembered (*N*D-RALUR) approaches based on anatomical indications. Clinical data, complications and outcomes were recorded.

**Results:**

Success rates improved significantly from 66% in Group A to 84% in Group B (*p* = 0.04). Postoperative vesicoureteral reflux occurred in 28% in Group A vs. 10% in Group B (*p* = 0.02). Complication rates decreased from 18% to 12%, with no conversions to open surgery in either group. The need for opioid analgesia was significantly lower in Group B (4% vs. 14%, *p* = 0.04). Our analysis showed a decreasing trend in both failure and complication rates, reflecting progressive improvement in surgical proficiency. RALUR was safely applied to increasingly complex cases, including redo surgeries and anatomical anomalies.

**Discussion:**

RALUR is a safe and effective technique for ureteral reimplantation in children, even in complex or redo cases. Surgical outcomes improved with experience, underscoring a manageable learning curve. The implementation of standardized techniques and increased surgeon expertise contributed to enhanced success rates and reduced morbidity. These findings support early integration of robotic training in pediatric urology and the broader adoption of RALUR in centers with appropriate expertise.

## 1 Introduction

Vesicoureteral reflux (VUR) and obstructive megaureter (OM) are common anomalies in pediatric urology. Although most patients benefit from conservative or endoscopic management, surgical intervention becomes necessary in cases of persistent high-grade reflux, recurrent febrile urinary tract infections (fUTIs), progressive renal impairment (evidenced by new scar formation or decline in renal function) or increasing hydroureteronephrosis (1).

The cross-trigonal open vesicoureteral reimplantation described by Cohen remains the gold standard for surgical correction, offering excellent long-terms results, with success rates exceeding 95% and low incidences of complications and reinterventions (1, 2). Nevertheless, the growing adoption of minimally invasive surgery (MIS) has led to increasing interest in robot-assisted laparoscopic ureteral reimplantation (RALUR). Although RALUR is not yet established as a first-line surgical option in current clinical guidelines, its use has expanded due to the well-recognized technical advantages of robotic platforms (1). These include tremor filtration, wristed instruments offering manual dexterity comparable to open surgery, and, high-resolution three-dimensional visualization that enhances depth perception—particularly useful in deep pelvic anatomy and in complex or redo cases (3, 4).

Originally developed for the treatment of VUR, RALUR is now increasingly applied to more anatomically complex scenarios, including duplex systems (DS), megaureters requiring tapering, ureteroceles, bladder diverticula, and redo surgeries. In these contexts, robotic assistance can improve surgical precision and reduce tissue trauma (5–8).

Reported outcomes for RALUR in the literature vary widely, with success rates ranging from 65% to 100%, and complication rates from 0% to 40%. These variations are attributed to case complexity, surgeon experience, and ongoing technical refinements over time (9–12). This variability highlights the critical role the surgical learning curve, with evidence suggesting improved outcomes after surpassing an initial threshold experience.

In this study, we report our experience with 100 consecutives pediatric RALUR procedures performed by a single surgeon. By comparing outcomes between the first and second groups of 50 cases, we aim to evaluate the impact of surgical experience on success rates, complications, and technical refinements over time.

## 2 Materials and methods

This prospective, single-center study was conducted over a five-year period, from May 2020 to May 2025. The study protocol was approved by the Regional Ethics Committee (Protocol RR2020 No. 567/2020). Written informed consent was obtained from the parents of all participating patients.

All pediatric patients who underwent RALUR at our Institution during the study period were eligible for inclusion. The study included patients diagnosed with either primary or iatrogenic OM, refluxing obstructive megaureter (ROM), or high-grade VUR. Complex anatomical conditions were also considered, such as those involving DS, ureteroceles, bladder diverticula, or patients requiring redo surgery following previous ureteral reimplantation or endoscopic procedures. In addition, selected cases of grade II–III VUR were included when associated with a poor response to endoscopic treatment, recurrent fUTIs, reflux nephropathy, or complex urinary tract anatomy.

Exclusion criteria included patients aged ≥18 years or follow-up duration of less than 6 months.

The choice between dismembered (D-RALUR) and non-dismembered (ND-RALUR) techniques was individualized based on etiology and anatomical findings. ND-RALUR was performed in cases of primary VUR with simple ureterovesical anatomy, whereas D-RALUR was reserved for those with OM, ROM, iatrogenic VUR following previous surgical or endoscopic uretero-vesical junction (UVJ) interventions, and in all cases with complex UVJ anatomy.

Data collection included patient demographics, clinical presentation, preoperative imaging findings, surgical details, intra-operative events, postoperative course, complications (graded using Clavien-Dindo classification) (13) and both short- and long-term outcomes. All patients underwent preoperative imaging evaluation. Renal and urinary tract ultrasonography (US) documented the anteroposterior pelvic diameter (APD), ureteral dilation, and presence of associated anomalies such as duplex systems, ureteroceles, or diverticula. Voiding cystourethrogram (VCUG) was performed to confirm presence and grade of VUR, bladder diverticula and ureterocele. Functional imaging, including either functional magnetic resonance urography (fMRU) or radionuclide studies (DMSA or MAG3), was performed to evaluate UVJ obstruction and determine differential renal function (DRF). All patients underwent standard blood and urine testing before surgery.

Each case was discussed within a multidisciplinary team comprising pediatric urologists, radiologists and nephrologists to confirm surgical indications and define the operative plan.

All patients underwent standardized postoperative follow-up with clinical and imaging evaluation at 1, 3, 6, and 12 months postoperatively, and annually thereafter. US was performed at each visit to assess APD and ureteral diameter. VCUG was reserved for cases presenting with recurrent fUTIs (≥2 episodes) or suggestive clinical and/or US signs of VUR. Functional imaging (fMRU or MAG3/DMSA) was repeated in cases of suspected UVJ obstruction or to monitor DRF stability.

Surgical success was defined as the absence of symptoms (fUTIs or flank pain) and the resolution or improvement of upper urinary tract dilatation on follow-up US. Importantly, symptoms and UTIs were only considered indicative of surgical failure when correlated with persistent or *de novo* VUR, as demonstrated by VCUG, or with evidence of UVJ obstruction, as shown by ultrasound and confirmed by MR urography. Surgical failure was defined as the presence of persistent or recurrent VUR, or evidence of UVJ obstruction requiring additional surgical or endoscopic intervention at the UVJ.

### 2.1 Surgical technique

**Figure 1:**
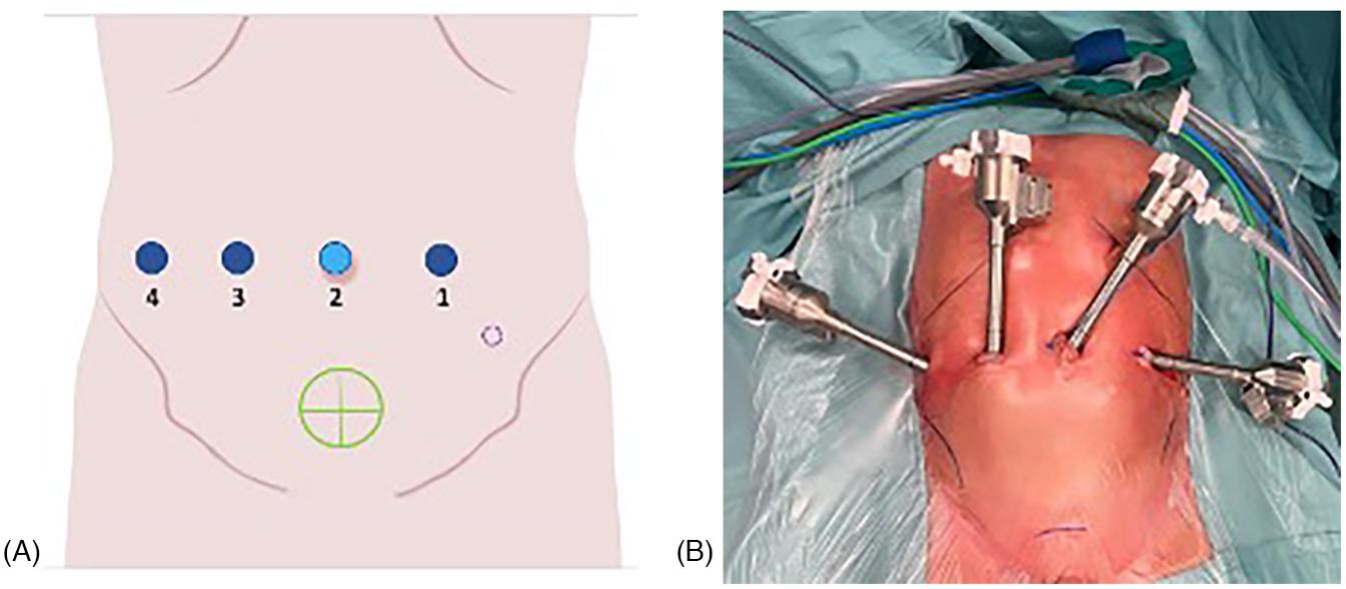
(**A,B)** Trocar placement for pediatric robotic ureteral reimplantation. Ports 1, 3, and 4 indicate the robotic tracer, port 2 corresponds to the camera trocar. The green circle marks the robotic target site, while the dashed purple circle represents the optional 3 mm accessory trocar used for placement of a double-J ureteral stent.

All procedures were performed by a single senior surgeon using the Da Vinci Xi® robotic surgical system. Patients were positioned supine with a 15°–20° Trendelenburg tilt. Following sterile preparation and draping, a transurethral bladder catheter was placed. The robotic system was docked on the patient's right side. A 0-degree endoscope was used for all procedures. Pneumoperitoneum was established via a trans umbilical 8 mm port using the Hasson technique, with intra-abdominal pressure maintained at 10 mmHg. Under direct vision, three additional 8 mm working ports were inserted along a horizontal line at the level of the umbilicus, ensuring a minimum 4 cm distance between ports (Figure 1). Usually, in small children, we adapt the distance between the trocars to the available surface area. In our experience, this is generally not associated with any conflicts between the robotic arms, and in any case, if such conflicts do arise, improving the clearance of the arms is usually sufficient to allow the procedure to proceed without issues.

**Figure 2:**
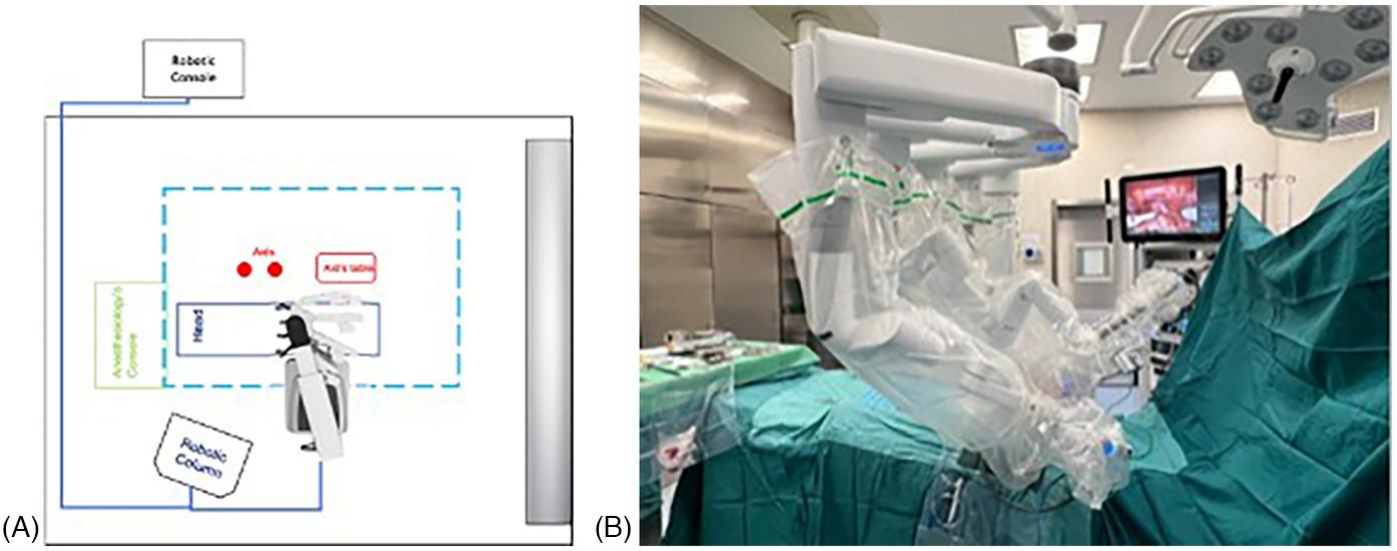
(**A,B)** Robot positioning. The robotic system approached from the patient's right side, with the docking site located in the pelvic region. The robotic column was positioned on the right side, at the level of the patient's head.

The robotic system was then docked (Figure 2).

RALUR was performed according to the Lich-Gregoir technique (14), incorporating the standardized LUAA technique (Length of detrusor tunnel, use of a U stich, placement of permanent ureteral Alignment suture and inclusion of ureteral Adventitia in detrusorraphy) described by Gundeti et al. (15). The distal ureter was dissected caudally across the iliac vessels to the UVJ, preserving the ureteral vascularization, the vas deferens or uterine artery. When needed, a vascular loop was applied to provide atraumatic traction of the ureter. An inverted “Y”-shaped detrusotomy was made, exposing the bladder mucosa. The submucosal tunnel was created in line with the ureter to prevent kinking or angulation, with a length of 4–5 cm. In D-RALUR cases, the ureter was excised distally and tailored, if necessary, as described by Starr (16) or tapered, as described by Hendren (17), before creating a new ureteral orifice close to the native meatus. A double-J stent was introduced through a robotic trocar or an assistant 3 mm port (placed in the left iliac fossa). The ureterovesical anastomosis was performed using interrupted 5-0 polydioxanone sutures. The ureter was then positioned within the detrusor tunnel, secured distally with a U-stitch anchoring the detrusor muscle at 5 and 7 o'clock around the ureteral adventitia. Detrusorraphy was performed using interrupted absorbable sutures, incorporating the ureteral adventitia at each stitch to prevent slippage, in a down-to-top fashion. To achieve a tension-free anastomosis, the bladder dome was mobilized toward the ureter and, when necessary, a psoas hitch was performed. In DS, both ureters were reimplanted within a shared tunnel. Finally, the bladder was refilled to check for urine leakage or ureteral kinking. A peri vesical drain was placed selectively based on intraoperative findings.

In the majority of cases, the typical postoperative management involves admission on the day of surgery. A negative urine culture, obtained within seven days before surgery, is required as part of the preoperative protocol. Postoperatively, a transurethral urinary catheter is left in place overnight. Oral intake is resumed on the same day, while intravenous hydration is maintained only for the first 24 h after the surgery. On postoperative day one, the catheter is removed and early ambulation is actively encouraged. In the absence of complications, patients are typically discharged on postoperative day two with as-needed analgesic therapy based on paracetamol. Upon discharge, we recommend a two-week break from sports activities.

### 2.2 Statistical analysis

Continuous variables were reported as medians with interquartile ranges (IQR), while categorical variables were presented as absolute counts and percentages. Associations between categorical variables were assessed using the chi-squared test, or the Fisher's exact test when appropriate. A *p*-value < 0.05 was considered statistically significant.

## 3 Results

Between May 2020 and May 2025, 103 RALUR procedures were performed in 99 patients. For this analysis, which required a minimum follow-up of six months, 100 procedures in 96 patients were included. The final three cases were excluded due to inadequate follow-up.

The cohort was divided into two groups in order to create two homogeneous groups in terms of quantity, making them more statistically comparable: Group A (the first 50 procedures) and Group B (the subsequent 50 procedures).

Group A comprised 14 females and 36 males, with a median age of 2.4 years (IQR = 5.5–1.6) and median body weight of 14.8 kg (IQR = 17.5–11.3). Group B included 10 females and 40 males, with a median age of 3.3 years (IQR = 6.9–1.1) and median body weight of 16 kg (IQR = 23–12).

The two patient groups were statistically comparable in terms of age (*p* = 0.09), weight (*p* = 0.38), and sex distribution (*p* = 0.48).

Demographic data are summarized in Table 1A.

**Table 1:** Demographic data.

(A)			
Demographic data	Median age (years)	Median weight (Kg)	Sex (M/F)
Group A	2.4 (IQR 5.5–1.6)	14.8 (IQR 17.5–11.3)	36/14
Group B	3.3 (IQR 6.9–1.1)	16 (IQR 23–12)	40/10
*P* value	*p* = 0.09	*p* = 0.38	*p* = 0.48

(B)		
Surgical indications	Group A	Group B
Vesical-ureteral reflux (VUR)	58% (29/50)	34% (17/50)
Primary obstructive megaureter	18% (9/50)	26% (13/50)
Secondary obstructive megaureter	14% (7/50)	18% (9/50)
Refluxing/obstructive megaureter	8% (4/50)	12% (6/50)
Others	2% (1/50)	4% (1/50)
(C)		
Anatomical anomalies	Group A	Group B
Double system	10% (5/50)	12% (6/50)
Ureterocele	2% (1/50)	12% (6/50)
Para-ureteral diverticulum	20% (12/50)	5% (10/50)
Total	50% (25/50)	58% (29/50)
(D)		
Previous ureteral surgeries	Group A	Group B
Endoscopic treatment of VUR (STING)	14	8
Open ureteral reimplantation + STING	5	7
Open ureteral reimplantation	1	3
Endoscopic incision of ureterocele	1	2
Endoscopic ureteral dilatation	0	3
Stent placement	0	2
Total	42% (21/50)	50% (25/50)

(A) Median age and weight and sex distribution (IQR in parentheses). The two groups are statistically comparable (*p* > 0.05). (B) Distribution of surgical indications. (C) Distribution of anatomical anomalies. (D) Previous ureteral surgeries performed.

The most common surgical indication was VUR, accounting for 58% of cases in Group A and 34% in Group B. This was followed by primary obstructive megaureter (18% vs. 26%), iatrogenic obstructive megaureter (14% vs. 18%), and refluxing/obstructive megaureter (8% vs. 12%) in Groups A and B, respectively. The remaining two cases underwent RALUR for rare conditions. Specifically, the one from group A underwent RALUR following UVJ avulsion incurred during ureteronephroscopy, while the other patient from group B underwent RALUR for a bladder urothelial adenoma involving the area oh the ureteral orifice (Table 1B).

Complex anatomical conditions were present in 25 patients (50%) in Group A and 29 patients (58%) in group B, with no statistically significant difference (*p* = 0.7).

The distribution of anatomical anomalies in each group is detailed in Table 1C.

A history of previous UVJ surgery was reported in 42% of patients in group A and 50% in Group B. In Group A, 14 patients had undergone at least one endoscopic treatment for VUR, 6 had prior open ureteral reimplantation (5 of whom had also received endoscopic treatment), and one had undergone endoscopic ureterocele resection.

In Group B, 10 patients had previous ureteral reimplantation (7 also had endoscopic reflux treatment), 8 had only undergone endoscopic VUR treatment and the remaining 7 had various prior endoscopic procedures including ureteral dilation, ureterocele resection, or stent placement (Table 1D).

No intraoperative complications occurred in either group. All patients had a urinary catheter placed intraoperatively and maintained postoperatively. The mean duration of bladder catheterization was 2.3 days in Group A (median 1.5 days; range, 1–12 days), with 54% of patients catheterized for only one day. In Group B, the mean duration was 1.6 days (median 1.5 days; range, 1–6 days), with 60% having catheter removal after one day (Table 2A). Unilateral JJ stent was placed intraoperatively in all D-RALUR cases, with median indwelling times of 45 days in Group A and 49 days Group B. No conversions to open surgery occurred in either group. The median length of hospital stay was comparable between the two groups (Table 2A).

**Table 2:** Data concerning perioperative details.

(A)					
	Median time of surgery (minutes)		Median duration of bladder catheterization (days)	Conversions	Median hospital stays (days)
	Console	Total			
Group A	95 (IQR 120–70)	137 (IQR 172–102)	1 (IQR 2–1)	0	2.5 (IQR 4–2)
Group B	84.5 (IQR 127.5–60)	136.5 (IQR 192–102)	1 (IQR 2.5–1)	0	3 (IQR 4–2)
(B)					
	Early complications		Opioid	NSAIDs	Paracetamol
	Clavien-Dindo 2	Clavien-Dindo 3b			
Group A	16% (8)	2% (1)	14% (7)	74% (37)	96% (48)
Group B	8% (4)	4% (2)	4% (2)	78% (39)	84% (42)
*p*-value	0.218	0.999	0.160	0.640	0.046

(A) Intraoperative and post-operative data. (B) Summary of postoperative early complications and analgesic use (range values are reported in parentheses).

Early postoperative complications (within 30 days) occurred in 9 patients (18%) in Group A. Among these, one was classified as Clavien-Dindo grade 3b and required a surgical intervention. The patient required surgical repair of an omental hernia at the robotic trocar site. The remaining 7 complications were managed conservatively; these included urinary tract infections and urinary leaks, which were addressed with intravenous antibiotic therapy and prolonged bladder catheterization, respectively. As a result, an increased length of hospital stay was observed.

In Group B, only 6 patients (12%) experienced early postoperative complications. Among these, 4 were Clavien-Dindo grade 2 (urinary tract infections or transient new-onset arterial hypertension) and were treated conservatively (Table 2B). Two patients required surgical repair of an omental herniation through the trocar site (*p* = 0.4).

Postoperative analgesic use differed between groups. In Group A, 14% of patients required opioids, 74% received non-steroidal anti-inflammatory drugs (NSAIDs), and 96% were administered paracetamol. In Group B, only 4% required opioids (for one day), 78% received NSAIDs, and 84% were treated with paracetamol, with 10% managed exclusively with paracetamol (Table 2B).

Complete resolution of the underlying disease was achieved in 66% of patients in Group A and 84% in Group B (*p* = 0.04, Table 3).

**Table 3:** Clinical outcomes and management of treatment failures in groups A and B.

Clinical outcomes	Group A	Group B	*p*-value (Chi-Square)
Resolution	66% (33/50)	84% (42/50)	0.038
VUR post RALUR	28% (14/50)	10% (5/50)	0.02
Endoscopic treatment	−24% (12/50)	−10% (5/50)	
Surgical redo	−4% (2/50)	−0% (0/50)	
Obstruction post RALUR	6% (3/50)	6% (3/50)	1
Endoscopic treatment	−4% (2/50)	−4% (2/50)	
Surgical redo	−2% (1/50)	−2% (1/50)	
Redo RALUR	6% (3/50)	2% (1/50)	0.3

Not all patients who experienced an UTI were found to have VUR on VCUG. Specifically, in Group A, 8 out of 9 patients with post-operative UTIs had confirmed VUR recurrence, whereas in Group B, only 6 out of 11 patients with UTIs showed VUR on VCUG.

Among the 34% (17/50) of Group A without resolution, 14 developed VUR: 12 successfully treated with endoscopic injection, and 2 requiring redo RALUR. Three patients developed post-operative UVJ obstruction; 2 were successfully managed with JJ stent placement, while 1 required a redo RALUR.

In Group B, 16% (8/50) did not achieve complete resolution. Five developed VUR, all of whom were managed successfully with endoscopic injection. Three patients showed postoperative UVJ obstruction: 2 were treated with stent placement, while 1 required a redo RALUR (Table 3).

All suspected cases of UVJ obstruction after RALUR were initially managed endoscopically by ureteral dilators, followed by placement of a ureteral stent. Surgical management (redo RALUR) was considered only in cases where obstruction persisted after stent removal, indicating failure of the endoscopic approach. Therefore, the treatment was not based on a predefined selection between endoscopic or surgical management; rather, all patients underwent an initial endoscopic attempt. In many cases, this approach was successful; in others, when it failed, redo RALUR was subsequently performed.

## 4 Discussion

Ureteral reimplantation is not universally considered a first-line treatment for distal ureteral pathologies. However, it becomes the preferred option in selected cases, particularly when endoscopic management fails or when anatomical complexities are present. Such complexities include ureterocele, para-ureteral diverticula, duplex collecting systems, or a history of previous surgical interventions involving the UVJ. In these scenarios, the likelihood of success with endoscopic approaches tend to decrease, making reconstructive surgery a more reliable and definitive option. Recent studies have reported favorable outcomes with endoscopic techniques for selected distal ureteral conditions. In particular, endoscopic balloon dilation for OM has shown success rates ranging from 85% to 90%, especially when performed in experienced centers. Similarly, the use of bulking agents for the endoscopic treatment of VUR has demonstrated resolution rates between 82% and 89% following a single injection, with cumulative success approaching 85% after repeated interventions. Despite these positive outcomes, endoscopic approaches are not universally effective, particularly in cases with underlying anatomical abnormalities or in patients who have undergone prior unsuccessful interventions. In such cases, ureteral reimplantation, whether performed via open, laparoscopic, or robotic-assisted technique, remains a well-established and effective surgical option (1, 18–21).

Ureteral reimplantation can be performed using various surgical techniques.

The open approach remains the most commonly used technique. Among open procedures, the Cohen reimplantation is the most commonly performed due to its excellent success rates. However, this method alters the anatomical course of the ureters by creating a cross-trigonal path, in which the ureters are tunneled to the contralateral side of the bladder. This issue is particularly relevant in patients with congenital anomalies of the kidney and urinary tract (CAKUT), who are at increased risk of stone formation later in life (22). The risk appears to be increased in individuals who have undergone ureteral reimplantation for VUR or OM. Recent evidence suggests that in post-Cohen patients requiring ureteral stenting or complex stone management, these procedures may be more technically demanding and associated with higher complication rates (2, 3, 5, 23, 24). In contrast, robot-assisted laparoscopic ureteral reimplantation (RALUR) preserves the ureter's natural linear course, potentially facilitating future interventions.

In cases of VUR, an extravesical approach with a Lich-Gregoire technique may be used. Success rates associated with the open approach for VUR are reported to range between 95% and 98% (23).

This technique has also been adapted to minimally invasive surgery, though current literature has yet to demonstrate clear superiority over the open approach. Moreover, laparoscopic ureteral reimplantation requires advanced surgical skills and is primarily limited to unilateral cases due to the ergonomic challenges associated with bilateral procedures (2, 5, 6, 19). Robotic surgery has enhanced the feasibility of minimally invasive approaches, offering a significantly shorter learning curve compared to conventional laparoscopy (5, 25). Additionally, the robotic platform allows for the performance of more complex procedures without increasing surgical difficulty (23).

RALUR outcomes do not seem to be adversely affected by the technical complexity introduced by previous endoscopic injections of bulking agents (26). In patients with a history of prior UVJ surgery, dense adhesions and poorly defined anatomical planes may be encountered in the abdominal cavity. In such cases, traditional laparoscopy makes ureteral isolation more challenging and carries a higher risk of injury and bleeding, whereas robotic surgery provides improved visualization and precision, thereby enhancing safety (27).

With the advancement of our surgical expertise, we progressively extended the indication for RALUR to include more complex cases. This trend is reflected in our patient cohorts, where we observed an increased proportion of individuals with anatomical anomalies and prior surgical history over time. Specifically, in Group A, RALUR was performed in 25 patients (50%) with anatomical anomalies and in 21 patients (42%) with a history of previous surgery. In contrast, Group B included 29 patients (58%) with anatomical anomalies and 25 patients (50%) with a surgical history (*p* = 0.4). These findings suggest a growing confidence in the application of RALUR to more challenging cases without compromising outcomes.

There are numerous studies addressing the learning curve (LC) in pediatric robot-assisted pyeloplasty, a procedure now widely regarded as one of the simplest and safest to perform using a robotic approach (28–32). In fact, the LC for laparoscopic pyeloplasty is significantly longer compared to the robotic approach. The literature also includes studies on LC of RALUR; however, since RALUR is not yet considered the gold standard, these studies remain of limited significance (13, 30).

The majority of studies assessing surgical learning curves have relied primarily on operative time as a surrogate marker for surgical experience, based on its presumed proportional relationship with technical proficiency. More recently, alternative methodologies have been introduced, incorporating a broader array of parameters—such as complication rates and overall clinical outcomes—to provide a more comprehensive and nuanced assessment of the learning process. Importantly, current evidence suggests that the initial stages of surgical training do not necessarily correlate with an increased incidence of complications or adverse outcomes. These findings underscore the safety of incorporating robotic-assisted procedures early in surgical training curricula and support their integration into standardized educational pathways (28–30).

In the present study, the overall success rate, defined as complete resolution following robotic ureteral reimplantation, was 75%, which is comparatively lower than success rates reported for alternative techniques in the existing literature. However, subgroup analysis revealed a statistically significant improvement in outcomes over time. Specifically, Group A exhibited a resolution rate of 66%, whereas Group B demonstrated a significantly higher rate of 84% (*p* = 0.04). Postoperative outcomes similarly differed between the two cohorts: the incidence of postoperative vesicoureteral reflux was markedly lower in Group B compared to Group A (6% vs. 28%), while the incidence of obstruction remained comparable between groups. Longitudinal analysis revealed a consistent trend of improvement in both surgical outcomes and complication rates, suggesting a progressive enhancement in technical proficiency. The observed increase in procedural success, coupled with a concurrent decline in adverse events, reflects the expected trajectory of a surgical learning curve. These findings highlight the correlation between increased surgical experience and improved patient outcomes, thereby reinforcing the procedural efficacy and safety as surgeon proficiency advances.

Management of unresolved cases differed between the groups, particularly concerning persistent VUR: in Group A, 86% of cases were managed endoscopically, while in Group B, 100% of cases resolved with endoscopic treatment alone (*p* = 0.3).

The lower resolution rate observed in our early cases likely reflects the relative novelty of robotic ureteral reimplantation and supports the potential for improvement as surgical experience grows. As noted in previous studies (33–35), both increased surgeon experience and the incorporation of technical refinements significantly influence success rates. The first surgeon had prior experience in both open and laparoscopic urologic procedures and is considered a senior, well-trained, and competent surgeon. While knowledge of alternative surgical approaches may provide a conceptual and technical foundation, in our opinion, it is helpful but not essential. Certainly, a more experienced surgeon may adapt more rapidly to robotic techniques, however, this is not guaranteed, as robotic proficiency is influenced by multiple factors beyond prior surgical background.

The current lack of standardization in robotic surgical technique may partially explain why success rates have not yet matched those of open procedures (23, 36).

With our experience, we have implemented several technical refinements. First, we performed a sagittal detrusor incision, fashioned in an inverted “Y” configuration, to expose the underlying mucosa and facilitate wrapping of the detrusor muscle around the UVJ, thereby avoiding excessive compression, as described by Gundeti et al. (15, 37).

It is essential to carefully align the UVJ with the axis of the detrusor incision to prevent ureteral angulation. Another important consideration is achieving an adequate submucosal tunnel length. In our experience, we observed a progressive increase in tunnel length, currently reaching an average of 4–5 cm. Importantly, we strive to tailor the tunnel length according to the distal ureteral diameter, thereby respecting the classic 5:1 length-to-diameter ratio to ensure effective antireflux function (38).

Additionally, during detrusorraphy, the ureteral adventitia was consistently incorporated into each stitch to ensure optimal tissue apposition, reducing the risk of ureteral slippage, within the submucosal tunnel (12, 39, 40).

Moreover, during robotic surgery, the vas deferens is better visualized and thus more easily preserved, whereas in the open Cohen technique it is at higher risk of injury, primarily due to limited direct visualization.

Complication rates following RALUR reported in the literature range between 10% and 12% (23, 27). In our series, the complication rate showed a progressive decline correlating with increased surgeon experience, decreasing from 30% to 12% (*p* = 0.02). One of the most commonly reported complications of extravesical robotic reimplantation is transient urinary retention, with incidence rates ranging from 0% to 37.5% (41).

In our cohort, no cases of acute postoperative urinary retention were observed, including in bilateral procedures. All patients received a Foley catheter postoperatively, which was maintained for an average of 2 days in Group A and 1 day in Group B. As previously described (12), we believe that this outcome can be achieved by limiting distal ureteral dissection to 1–5 cm, maintaining proximity to the adventitial layer, and avoiding the use of electrocautery (15).

Notably, we observed no conversions to open surgery in our series, supporting the feasibility and safety of the robotic approach, even in the smallest patients, those under one year of age and weighing less than 10 kg.

These results are consistent with those reported by other centers that have published their experience with robotic ureterovesical reimplantation (42).

Our study is not without limitations, including variability in the surgical procedures and patient characteristics, as well as technical adjustments made during the learning curve. Nevertheless, our findings are promising, demonstrating that robotic surgery of the UVJ is a feasible and safe procedure in pediatric patients, including those of low body weight, younger age, and in technically challenging cases.

## 5 Conclusions

RALUR represents a relatively recent surgical innovation that has yet to achieve widespread standardization. While our current success rates have not fully reached those reported for traditional open procedures, RALUR demonstrates significant promise, especially in light of its comparatively shorter and more manageable learning curve. Our data highlight the positive correlation between growing surgical expertise in robotic techniques and enhanced patient outcomes, thereby reinforcing the importance of ongoing refinement and optimization of this approach. Given its novelty, RALUR still offers considerable potential for further technical improvements and clinical advancements.

## Data Availability

The raw data supporting the conclusions of this article will be made available by the authors, without undue reservation.

## References

[1] EAU Guidelines on Paediatric Urology. Presented at the EAU Annual Congress Paris 2024. ISBN 978-94-92671-23-3. Available online at: https://uroweb.org/guidelines/paediatric-urology (Accessed December 16, 2024).

[2] Gerber JA Koh CJ. Robot-assisted laparoscopic ureteral reimplantation in children: a valuable alternative to open surgery. World J Urol. (2020) 38:1849–54. 10.1007/s00345-019-02766-y 31004205

[3] Peters CA. Robotically assisted surgery in pediatric urology. Urol Clin North Am. (2004) 31:743–52. 10.1016/j.ucl.2004.06.007 15474601

[4] Satyanarayan A Peters CA. Advances in robotic surgery for pediatric ureteropelvic junction obstruction and vesicoureteral reflux: history, present, and future. World J Urol. (2020) 38:1821–6. 10.1007/s00345-019-02753-3 30953140

[5] Esposito C Masieri L Fourcade L Ballouhey Q Varlet F Scalabre A Pediatric robot-assisted extravesical ureteral reimplantation (revur) in simple and complex ureter anatomy: report of a multicenter experience. J Pediatr Urol. (2023) 19:136.e1–e7. 10.1016/j.jpurol.2022.10.024 36344364

[6] Mittal S Srinivasan A Bowen D Fischer KM Shah J Weiss DA Utilization of robot-assisted surgery for the treatment of primary obstructed megaureters in children. Urology. (2021) 149:216–21. 10.1016/j.urology.2020.10.015 33129867

[7] Arlen AM Broderick KM Travers C Smith EA Elmore JM Kirsch AJ. Outcomes of complex robot-assisted extravesical ureteral reimplantation in the pediatric population. J Pediatr Urol. (2016) 12:169.e1–e6. 10.1016/j.jpurol.2015.11.007 26747012

[8] Sforza S Cini C Negri E Bortot G Di Maida F Cito G Ureteral reimplantation for primary obstructive megaureter in pediatric patients: is it time for robot-assisted approach? J Laparoendosc Adv Surg Tech. (2022) 32:231–6. 10.1089/lap.2021.0246 34905408

[9] Essamoud S Ghidini F Andolfi C Gundeti MS. Robot-assisted laparoscopic extravesical ureteral reimplantation (RALUR-EV): a narrative review. Transl Pediatr. (2024) 13:1634–40. 10.21037/tp-23-336 39399704 PMC11467227

[10] Esposito C Castagnetti M Autorino G Coppola V Cerulo M Esposito G Robot-assisted laparoscopic extra-vesical ureteral reimplantation (ralur/revur) for pediatric vesicoureteral reflux: a systematic review of literature. Urology. (2021) 156:e1–e11. 10.1016/j.urology.2021.06.043 34324913

[11] Baek M Koh CJ. Lessons learned over a decade of pediatric robotic ureteral reimplantation. Investig Clin Urol. (2017) 58:3–11. 10.4111/icu.2017.58.1.3 28097262 PMC5240282

[12] Mattioli G Fanti F Carlucci M Parodi S Fiorenza Vl. From open to robotic surgery in pediatric ureteral reimplantation: overcoming the learning curve for improved outcomes. Front Surg. (2025) 12:1573233. 10.3389/fsurg.2025.1573233 40364919 PMC12069337

[13] Dindo D Demartines N Clavien PA. Classification of surgical complications: a new proposal with evaluation in a cohort of 6336 patients and results of a survey. Ann Surg. (2004) 240(2):205–13. 10.1097/01.sla.0000133083.54934.ae 15273542 PMC1360123

[14] Gregoir W Vanregemorter G. Congenital vesico-ureteral reflux. Urol Int. (1964) 18:122–36. 10.1159/000279233 14215746

[15] Gundeti MS Boysen WR Shah A. Robot-assisted laparoscopic extravesical ureteral reimplantation: technique modifications contribute to optimized outcomes. Eur Urol. (2016) 70(5):818–23. 10.1016/j.eururo.2016.02.065 27036858

[16] Starr A. Ureteral plication. A new concept in ureteral tailoring for megaureter. Invest Urol. (1979) 17(2):153–8. 468516

[17] Hendren WH. Operative repair of megaureter in children. J Urol. (1969) 101(4):491–507. 10.1016/S0022-5347(17)62370-X), 5776032

[18] Doudt AD Pusateri CR Christman MS. Endoscopic management of primary obstructive megaureter: a systematic review. J Endourol. (2018) 32(6):482–7. 10.1089/end.2017.0434 29676162

[19] Elder JS Diaz M Caldamone AA Cendron M Greenfield S Hurwitz R Endoscopic therapy for vesicoureteral reflux: a meta-analysis. I. Reflux resolution and urinary tract infection. J Urol. (2006) 175(2):716–22. 10.1016/S0022-5347(05)00210-7 16407037

[20] Läckgren G Cooper CS Neveus T Kirsch AJ. Management of vesicoureteral reflux: what have we learned over the last 20 years? Front Pediatr. (2021) 9:650326. 10.3389/fped.2021.650326 33869117 PMC8044769

[21] Faruque A Delaney D. Endoscopic management of primary obstructed megaureter: is it justified? Literature review. J Pediatr Urol. (2020) 16:S14–5. 10.1016/j.jpurol.2020.05.043

[22] Turan H Şimşek ÖÖ. Overview of microlithiasis in infancy in pediatric urology. J Pediatr Res. (2025) 12(1):27–30. 10.4274/jpr.galenos.2025.86300

[23] Mattioli G Lena F Fiorenza V Carlucci M. Robotic ureteral reimplantation and uretero-ureterostomy treating the ureterovesical junction pathologies in children: technical considerations and preliminary results. J Robotic Surg. (2022) 17(2):659–67. 10.1007/s11701-022-01478-7 36287349

[24] Torii K Hamamoto S Taguchi K Okada S Inoue T Isogai M Efficacy of mini-endoscopic combined intrarenal surgery for pediatric kidney calculi: a single center retrospective study. Sci Rep. (2024) 14(1):17134. 10.1038/s41598-024-68258-1 39054390 PMC11272918

[25] Krebs TF Schnorr I Heye P Häcker FM. Robotically assisted surgery in children-a perspective. Children (Basel). (2022) 9(6):839. 10.3390/children9060839 35740776 PMC9221697

[26] Comez I Ucar T Telli O Gunaydin B Silay MS. Does previous endoscopic subureteric injection (STING) effect the outcomes of robot-assisted laparoscopic ureteral reimplantation surgery (RALUR) in children? J Pediatr Urol. (2023) 19(6):800.e1–e6. 10.1016/j.jpurol.2023.07.013 37607849

[27] Wu Q Yang P Liang Y Qin H Gan Z Qin B. Comparative outcomes of robot-assisted vs traditional laparoscopic ureteral reimplantation for lower ureteral stenosis: a single center study. Med Sci Monit. (2025) 31:e946803. 10.12659/MSM.946803 40158194 PMC11967284

[28] Pakkasjärvi N Krishnan N Ripatti L Anand S. Learning curves in pediatric robot-assisted pyeloplasty: a systematic review. J Clin Med. (2022) 11(23):6935. 10.3390/jcm11236935 36498510 PMC9737296

[29] Planchamp T Bento L Mouttalib S Belbahri I Coustets B Aissa DA Robotic pyeloplasty learning curve for a pediatric surgeon without previous laparoscopic pyeloplasty experience. J Robot Surg. (2023) 17(6):2955–62. 10.1007/s11701-023-01737-1 37864128

[30] Kim JK Batra N Shavnore R Szymanski KM Misseri R Kaefer M Attaining competency and proficiency in pediatric robot-assisted laparoscopic ureteric reimplantation: a learning curve configuration using cumulative sum analysis. World J Urol. (2025) 43(1):372. 10.1007/s00345-025-05658-6 40515856 PMC12167274

[31] Kim JK Batra NV Szymanski KM Shavnore R Misseri R Kaefer M Evaluating the trainee impact on robotic surgery learning curves: a CUSUM analysis of large series pediatric robot-assisted laparoscopic pyeloplasty. J Robot Surg. (2025) 19(1):119. 10.1007/s11701-025-02281-w 40106060

[32] Zhou L Huang J Xie H Chen F. The learning curve of robot-assisted laparoscopic pyeloplasty in children. J Robot Surg. (2024) 18(1):97. 10.1007/s11701-024-01856-3 38413450

[33] Boysen WR Akhavan A Ko J Ellison JS Lendvay TS Huang J Prospective multicenter study on robot-assisted laparoscopic extravesical ureteral reimplantation (RALUR-EV): outcomes and complications. J Pediatr Urol. (2018) 14(3):262.e1–e6. 10.1016/j.jpurol.2018.01.020 29503220

[34] Boysen WR Ellison JS Kim C Koh CJ Noh P Whittam B Multi-institutional review of outcomes and complications of robot-assisted laparoscopic extravesical ureteral reimplantation for treatment of primary vesicoureteral reflux in children. J Urol. (2017) 197(6):1555–61. 10.1016/j.juro.2017.01.062 28130103

[35] Sahadev R Spencer K Srinivasan AK Long CJ Shukla AR. The robot-assisted extravesical anti-reflux surgery: how we overcame the learning curve. Front Pediatr. (2019) 7(93). 10.3389/fped.2019.00093 30984718 PMC6450052

[36] Elbakry AA Abdelhalim A Al-Omar O. Tips and tricks for the extravesical robotic-assisted laparoscopic ureteral reimplantation for pediatric vesicoureteral reflux. J Pediatr Urol. (2023) 19(6):816–7. 10.1016/j.jpurol.2023.07.014 37524572

[37] Hajiyev P Sloan M Fialkoff J Gundeti MS. The LUAA gundeti technique for bilateral robotic ureteral reimplantation: lessons learned over a decade for optimal (resolution, urinary retention, and perioperative complications) trifecta outcomes. Eur Urol Open Sci. (2023) 57:60–5. 10.1016/j.euros.2023.09.006 37790798 PMC10543781

[38] Levine BA Papastamatiou J Ehrlich RM. The length of the submucosal ureteral tunnel in prevention of vesicoureteral reflux. J Urol. (1969) 101(2):211–4.

[39] Perovic S. Surgical treatment of megaureters using detrusor tunneling extravesical ureteroneocystostomy. J Urol. (1994) 152(2 Pt 2):622–5. discussion 626–7. 10.1016/s0022-5347(17)32666-6 8021984

[40] Kim SW Lim NL Lee YS Han SW Im YJ. Laparoscopic intravesical detrusorrhaphy with ureteral plication for megaureter: a novel technique. Urology. (2015) 86(1):187–91. 10.1016/j.urology.2015.02.023 26142606

[41] Saldivar RM Johnston AW Roth JD. Bladder dysfunction after ureteral reimplantation. Curr Bladder Dysfunct Rep. (2022) 17(3):169–78. 10.1007/s11884-022-00658-3

[42] Zhang X Tao T Li P Zhao Y Cao H Tao Y Comparison of robot-assisted laparoscopic extravesical ureteral reimplantation for primary vesicoureteral reflux in infants under one year of age and older children. J Pediatr Surg. (2025) 60(3):162114. 10.1016/j.jpedsurg.2024.162114 39740279

